# Cancer-Associated Fibroblasts and Tumor Cells in Pancreatic Cancer Microenvironment and Metastasis: Paracrine Regulators, Reciprocation and Exosomes

**DOI:** 10.3390/cancers14030744

**Published:** 2022-01-31

**Authors:** Yoshiaki Sunami, Johanna Häußler, Anais Zourelidis, Jörg Kleeff

**Affiliations:** Department of Visceral, Vascular and Endocrine Surgery, Martin-Luther-University Halle-Wittenberg, University Medical Center Halle, 06120 Halle, Germany; johanna.haeussler@uk-halle.de (J.H.); anais.zourelidis@uk-halle.de (A.Z.); joerg.kleeff@uk-halle.de (J.K.)

**Keywords:** pancreatic cancer, cancer-associated fibroblasts, tumor microenvironment, paracrine signals, reciprocal signals, exosomes, pre-metastatic niche

## Abstract

**Simple Summary:**

Cancer-associated fibroblasts in the stromal tumor microenvironment play a key role in cancer progression, invasion, metastasis, and therapy resistance. Cancer-associated fibroblasts communicate with tumor cells through diverse factors, such as growth factors, hedgehog proteins, cytokines, and chemokines, regulating signaling activity in paracrine as well as paracrine-reciprocal ways. Furthermore, cancer-associated fibroblasts, not only tumor cells, secrete exosomes that drive pre-metastatic niche formation and metastasis.

**Abstract:**

Pancreatic cancer is currently the fourth leading cause of cancer deaths in the United States, and the overall 5 year survival rate is still only around 10%. Pancreatic cancer exhibits a remarkable resistance to established therapeutic options such as chemotherapy and radiotherapy, in part due to the dense stromal tumor microenvironment, where cancer-associated fibroblasts are the major stromal cell type. Cancer-associated fibroblasts further play a key role in cancer progression, invasion, and metastasis. Cancer-associated fibroblasts communicate with tumor cells, not only through paracrine as well as paracrine-reciprocal signaling regulators but also by way of exosomes. In the current manuscript, we discuss intercellular mediators between cancer-associated fibroblasts and pancreatic cancer cells in a paracrine as well as paracrine-reciprocal manner. Further recent findings on exosomes in pancreatic cancer and metastasis are summarized.

## 1. Introduction

Pancreatic cancer is currently the fourth leading cause of cancer deaths in the United States, and its incidence continues to increase in both females and males [1]. Pancreatic cancer will most probably be the second most common cause of cancer death by 2030 [2]. The prognosis remains very poor, and the overall 5 year survival rate is still only around 10%, despite recent therapeutic advances such as more effective palliative, adjuvant, and neo-adjuvant chemotherapies and more radical and safer surgery [1,3]. A hallmark of pancreatic cancer is its remarkable resistance to established therapeutic options such as chemotherapy and radiotherapy, in part due to the dense stromal tumor microenvironment, where cancer-associated fibroblasts (CAF) are the major stromal cell type [4,5]. The molecular crosstalk between cancer cells and CAFs is crucial for tumor progression and metastatic process. Tumor cells communicate with CAFs not only through paracrine as well as paracrine-reciprocal signaling mechanisms, but also through exosomes [5,6]. In the current manuscript, we discuss intercellular mediators between CAFs and pancreatic cancer cells in a paracrine as well as a paracrine-reciprocal manner. Further, recent findings on exosomes in pancreatic cancer and metastasis are summarized.

## 2. Growth Factors Act as Paracrine Signals between Cancer-Associated Fibroblasts and Pancreatic Cancer Cells

**Figure 1:**
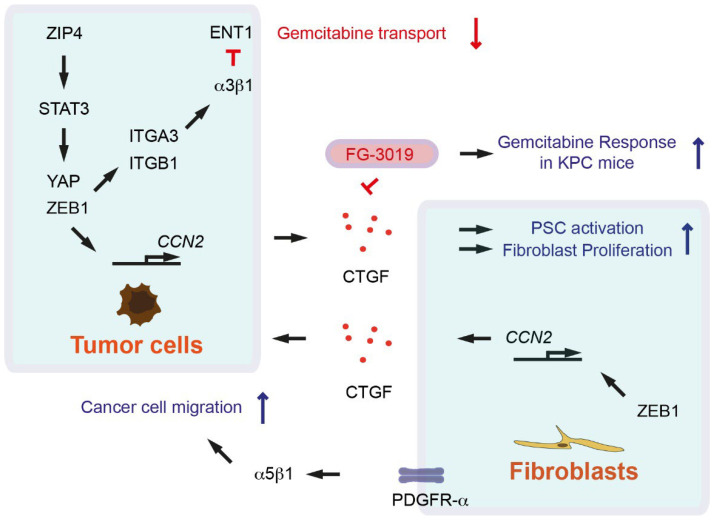
Growth factors as paracrine factors for cancer-associated fibroblasts (CAFs) and tumor cells. Arrows indicate activation or induction symbols. The inhibition symbols are colored in red.

Resident fibroblasts and especially pancreatic stellate cells (PSCs) are major sources of CAFs in pancreatic cancer, but also characterized by their diverse origins [5,7,8,9]. CAFs and activated PSCs produce several growth factors such as connective tissue growth factor (CTGF); epidermal growth factor (EGF); platelet-derived growth factor (PDGF); and inflammatory cytokines, chemokines, and extracellular matrix (ECM) proteins that promote cancer cell proliferation, therapy resistance, and immune escape [7,10]. CTGF is known to participate in neoplastic cell-stroma interactions in cancer. CTGF is highly expressed in CAFs and tumor cells in pancreatic cancer mouse model called KPC (*Pdx1-Cre*; *lox-stop-lox-Kras^G12D/+^*; *Trp53^R172H/+^*) (Table 1) [11]. Inhibition of CTGF with the monoclonal antibody FG-3019 enhances gemcitabine chemotherapy response without increasing gemcitabine concentrations in KPC mice (Figure 1) [11]. CTGF inhibition with FG-3019 does not lead to reduction of stromal contents; rather, it alters tumor cell survival as well as tumor-stromal interactions [11]. Treatment with another CTGF-neutralizing antibody FG-3154 suppresses PSC activation caused by repeated cerulein injection as an established pancreatitis model [12]. In cholangiocarcinoma, overexpression of the transcription factor zinc finger E-box-binding homeobox 1 (ZEB1) in tumor cells leads to increased CTGF expression. Culture medium from ZEB1-overexpressing tumor cells induces proliferation of myofibroblasts (Figure 1) [13]. In human cholangiocarcinoma, ZEB1 is expressed in CAFs, correlating with cellular communication network factor 2 (*CCN2*) gene (encoded for CTGF) expression (Figure 1) [13]. ZEB1 regulates the expression of paracrine signals such as hepatocyte growth factor (HGF) and interleukin 6 (IL-6) in tumor cells and CAFs, highlighting that the ZEB1–CTGF axis plays a key role in regulating the network between tumor cells and CAFs [13]. Dysregulation of phosphatidylinositol-4,5-bisphosphatase 3-kinase (PI3K) and Hippo signaling pathways synergistically induce Hippo effector YES-associated protein (YAP), which in turn upregulates CTGF for PSC activation (Figure 1) [12]. ZEB1 directly interacts with YAP, where ZEB1 turns into a transcriptional activator and regulates *CCN2* gene expression (Figure 1) [14]. Zinc/iron-regulated transporter-like protein ZIP4 (encoded by solute carrier family 39 member 4 *SLC39A4* gene) activates STAT3, which subsequently induces ZEB1. ZEB1 in turn activates the expression of integrin ITGA3 and ITGB1. Integrin α3β1 signaling inhibits the expression of gemcitabine transporter equilibrative nucleoside transporter 1 (ENT1, encoded by solute carrier family 29 member 1 *SLC29A1*) (Figure 1) [15]. ZIP4 also upregulates YAP1, and ZEB1-YAP1-containing complex activates *ITGA3* transcription. ZEB1-YAP1 co-activation promotes pancreatic cancer metastasis as well as epithelial-to-mesenchymal transition (EMT) plasticity. EMT plasticity is a dynamic and reversible transition between EMT and mesenchymal-to-epithelial transition (MET) [16]. It has also been suggested that CTGF secreted from pancreatic cancer cells binds to integrin α5β1 and promotes proliferation, adhesion, and migration of PSCs [17]. In summary, CTGF acts as a key paracrine mediator in signal transduction networks derived from both CAFs and tumor cells.

EGF receptor signaling is important for pancreatic intraepithelial neoplasia (PanIN) and pancreatic cancer development induced by oncogenic KRAS [25,26]. Administration of 5-fluorouracil (5-FU)-incorporated EGFR receptor-targeted aptamers attenuates pancreatic cancer development in a pancreatic cancer mouse model (*Ptf1a-Cre*; *lox-stop-lox-Kras^G12D/+^*; *Trp53^lox/+^*) [27]. A potent epigenetic regulator lysine (K) demethylase 3A (KDM3A) regulates expression of EGFR through Krueppel-like factor 5 (KLF5) and mother against decapentaplegic homolog 4 (SMAD4). CRISPR-mediated ablation of *KLF5*, *SMAD4*, or *EGFR* in pancreatic tumor cells leads to increased T-cell infiltration and improved combination immunotherapy response with gemcitabine, abraxane, CD40 agonistic antibody, CTLA4-blocking antibody, and PD-1-blocking antibody (named as GAFCP) [28]. Loss of *SMAD4* is observed frequently in pancreatic cancer patients and has been considered as a tumor suppressor gene [4]. The discrepant observation—tumor suppressor or tumor promotor—may be due to the different roles of SMAD4 during tumor initiation vs. tumor progression [28].

With cancer cells isolated from KPC mice, it has been demonstrated that *Trp53* mutations drive pancreatic cancer metastasis through PDGF receptor β (PDGFR-β) signaling [29]. Activation of PDGFR-β signaling by p53 mutations leads to expression of a small GTPase ADP ribosylation factor 6 (ARF6) and its downstream effector ArfGAP with SH3 domain, ankyrin repeat, and PH domain (ASAP1, also known as AMAP1), which drives PD-L1-mediated immune evasion [30]. Pancreatic cancer patients with tumors harboring high PDGFR-β and nuclear p53 protein expression exhibit a high incidence of metastasis and shorter postoperative survival [31]. PDGFR-β has also been suggested to be a marker of CAFs and activated PSCs. High stromal expression of PDGFR-β is associated with shorter overall survival of pancreatic cancer patients [32]. In another study, it has been demonstrated that CAFs overexpress PDGFR-α, which increases contractility leading to migration of cancer cells via αv integrins. Inhibition of PDGFR-α abrogates α5β1 integrin activity and changes in matrix organization, namely, from aligned fibers to more random organization (Figure 1) [33]. Taken together, growth factors can play key roles in cancer cell growth and metastasis in autocrine but also in paracrine manner between cancer cells and CAFs.

## 3. Sonic Hedgehog and Insulin Signaling as Paracrine and Paracrine-Reciprocal Signals between Cancer-Associated Fibroblasts and Pancreatic Cancer Cells

**Figure 2:**
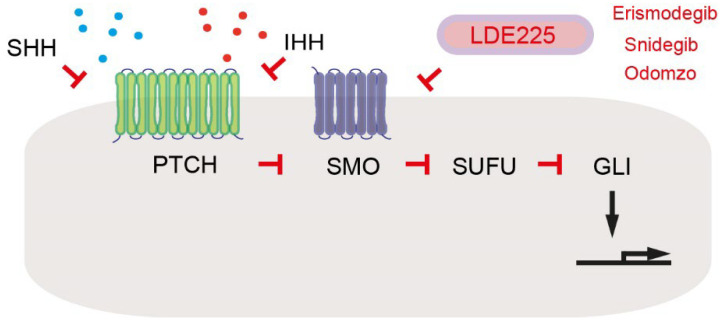
Hedgehog signaling. Arrows indicate activation or induction. Inhibition symbols are colored in red.

In the inducible KRAS pancreatic cancer mouse model (*Ptf1-Cre; Rosa26-rtTa*; *TetO-Kras^G12D^*), it has been described that pancreatic tumor cells regulate PSCs non-cell autonomously by secreting factors including Sonic hedgehog (SHH) [18]. HH ligands, such as SHH and Indian hedgehog (IHH), bind to the extracellular domain of patched 1 (PTCH) (Figure 2). This ligand–receptor complex loses repressive effect of Smoothened (SMO). Active SMO inhibits suppressor of fused homolog (SUFU), leading to release and activation of glioma-associated oncogene homolog (GLI) transcription factors, where *GLI1* itself is a part of transcriptional targets (Figure 2) [34]. SHH from tumor cells in turn induces expression of growth factors insulin growth factor 1 (IGF1) and growth arrest-specific 6 (GAS6) in PSCs. IGF1 and GAS6 act as paracrine-reciprocal signals for activating the receptor tyrosine kinases IGF1R and AXL (“anexelenko”, which means “uncontrolled” in Greek [35]), respectively, in tumor cells (Table 1) [18]. IGF1R and AXL activate Akt signaling and increase mitochondrial performance, proliferation, and resistance to apoptosis in tumor cells [18]. Pharmacological inhibition of the Hedgehog pathway in CAFs with a SMO antagonist LDE225 attenuates expression of a Hedgehog target *Gli1* in the fibroblast compartment as well as pancreatic cancer growth in KPC mice [36]. LDE225, also known as erismodegib, sonidegib, or Odomzo, is an FDA-approved SMO antagonist for treating cancer patients (Figure 2) [37]. Taken together, deletion of SHH or SMO commonly inhibits tumor growth. However, conditional deletion of *Shh* results in more aggressive, undifferentiated tumors in a pancreatic cancer mouse model (*Pdx1-Cre*; *lox-stop-lox-Kras^G12D/+^*; *Trp53^lox/+^*; *Shh^lox/lox^*; *lox-stop-lox-YFP-Rosa26*) [38]. Furthermore, treatment with LDE225 leads to a reduction of PDPN-positive, α-smooth muscle actin (α-SMA)-positive myofibroblastic CAFs (myCAFs) but an expansion of inflammatory CAFs (iCAFs) in KPC mice. Although the treatment with LDE225 attenuates pancreatic cancer growth in KPC mice, LDE225 increases chemokine *Cxcl12* expression, reduces CD8-positive T cells, and increases regulatory T cell (Treg) immunosuppression [36]. It has been shown that genetic ablation of *Smo* in fibroblast-specific protein 1 (FSP1)-positive cells increases acinar-to-ductal metaplasia (ADM) in an oncogenic KRAS mouse model (*Mist-Kras^G12D/+^*; *Fsp-Cre*; *Smo^lox/−^*) [39]. The role of Hedgehog signaling in pancreatic cancer is complex. Further studies are needed to unveil critical differences between SHH and IHH for activation of the Hedgehog signaling pathways in cell type-dependent manners.

## 4. Cancer-Associated Fibroblast Subtypes and Paracrine Factors

The myCAFs and iCAFs are well-described subtypes of CAFs, which co-exist both in pancreatic cancer patients and in KPC mice [40]. When PSCs are co-cultured with organoids derived from KPC mice, the cells differentiate into myCAFs and iCAFs [40]. It has been identified that myCAF subtype exhibits elevated fibroblast-activation protein α (FAP) and α-SMA. The majority of fibroblasts in human pancreatic tumors and in tumors from KPC mice express FAP but low levels of α-SMA [40]. Besides *Acta2* (coding α-SMA), several genes such as *Col1a1*, *Col5a1*, *Col6a1*, *Ctgf*, and *Vim* are upregulated in myCAFs [40]. The iCAF subtype expresses cytokines IL-6, IL-11, and leukemia inhibitory factor (LIF) with low α-SMA expression. Several chemokines such as *Cxcl1* and *Cxcl2* are upregulated in iCAFs, but not in the myCAF subpopulation [40]. Indirect co-culture of quiescent PSCs with organoids derived from pancreatic cancer patients leads to differentiation of PSCs into iCAFs [40]. IL-1α from tumor cells activates nuclear factor kappa-light-chain-enhancer of activated B cells (NF-κB) signaling and expression of LIF in iCAFs [19]. LIF activates Janus kinase (JAK)/STAT signaling in iCAFs and establishes an autocrine positive feedback loop by upregulating expression of IL-1 receptor type 1 (IL-1R1) (Table 1) [19]. Further, LIF from CAFs can act as a key paracrine(-reciprocal) factor for activating signal transducer and activator of transcription 3 (STAT3) signaling in cancer cells [41]. LIF receptor and its co-receptor IL-6 signal transducer (IL6ST, gp130) interact with STAT3 in human pancreatic cancer cells stimulated with CAF-conditioned medium [41]. Conditional deletion of *Lifr* reduces pancreatic tumor progression, but not ADM formation or tumor initiation in an oncogenic KRAS mouse model (*Pdx1-Cre*; *lox-stop-lox-Kras^G12D/+^*; *Trp53^lox/lox^*; *Lifr^lox/lox^*; *lox-stop-lox-Rosa26^Luc/Luc^*). Conditional deletion of *Lifr* increases overall survival, which is further prolonged by gemcitabine administration, suggesting that LIFR signaling plays a role in gemcitabine chemoresistance [41]. Consistently, administration of LIF-neutralizing antibody also increases overall survival and gemcitabine chemoresistance [41]. A soluble recombinant variant of extracellular domain of human LIFR binds and sequesters human LIF for inhibiting LIFR signaling. Treatment with the variant attenuates tumor growth in a human pancreatic tumor cell xenograft [42]. LIF expression is associated with shorter overall survival and recurrence-free survival in pancreatic cancer patients. Increased serum LIF is a biomarker to predict lymph node metastasis and distant metastasis in pancreatic cancer patients [43]. Another study, however, showed that expression of LIFR was associated with longer overall survival of pancreatic cancer patients [44]. Oncogenic KRAS downregulates LIFR. Downregulation of LIFR is important for KRAS-mediated neoplastic transformation [44]. Wang et al. showed that oncogenic KRAS induced LIF expression in pancreatic cancer cells, where the mitogen-activated protein kinase kinase (MEK)/extracellular signal-regulated kinase (ERK) signaling is essential [45]. Treatment with LIF, but not IL-6, subsequently activates YAP/Tafazzin (TAZ, phospholipid-lysophospholipid transacylase)/TEA domain transcription factor (TEAD)-dependent transcription [45]. Further clarification is needed to understand the precise role of LIF/LIFR signaling in pancreatic cancer.

## 5. Chemokines as Paracrine and Paracrine-Reciprocal Factors Secreted by Cancer-Associated Fibroblasts and Tumor Cells

**Figure 3:**
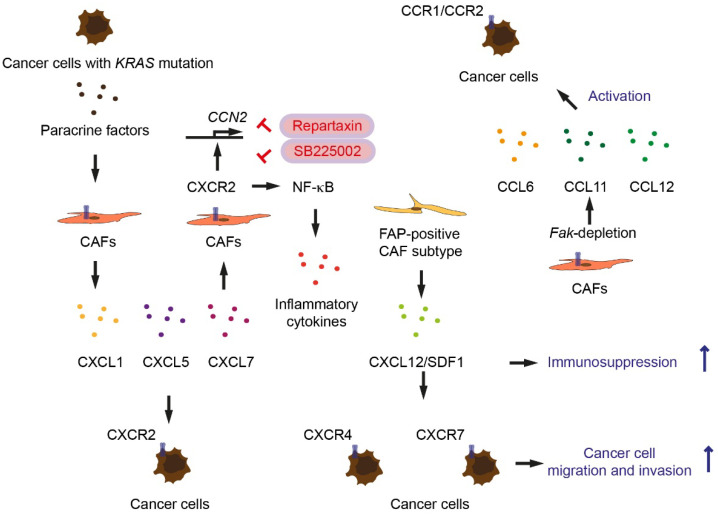
Chemokines and chemokine receptors as paracrine and paracrine-reciprocal factors for cancer-associated fibroblasts (CAFs) and tumor cells. Arrows indicate activation or induction. Inhibition symbols are colored in red.

Oncogenic KRAS upregulates C-X-C motif chemokine receptor CXCR2 [20]. CXCL1, CXCL2, CXCL3, CXCL5, CXCL7, and CXCL8 are ligands for CXCR2 [46]. CXCL1 is highly expressed in human pancreatic cancer patient specimens [47]. Expression of CXCL1 is dependent on receptor-interacting protein 1 (RIP1) and RIP3, key regulators for necroptosis (programmed necrosis), which are also highly expressed in human pancreatic cancer [47]. In a KRAS-induced pancreas cell orthotopic implantation model, *Rip3* deletion (*Kras^G12D/+^*; *Rip^−/−^*) leads to an increased number of T cells and decreased number of tumor-associated macrophages (TAM), indicating that necroptosis-induced CXCL1 signaling promotes immunosuppression [47]. Treating CAFs with conditioned media of cells derived from KC mice (*Pdx1-Cre*; *lox-stop-lox-Kras^G12D/+^*) increases *Cxcl2* and *Cxcl7* expression [20]. Treatment with KC-conditioned media on CAFs induces secretion of CXCL1, CXCL5, and CXCL7 greater than treatment with conditioned media from *KRAS* wild-type pancreatic cancer cells (Table 1) (Figure 3) [20]. These data suggest that KRAS-driven factors from pancreatic cells act on CAFs, inducing secretion of CXCR2 ligands. The CXCR2 ligands may reciprocally activate CXCR2 signaling in tumor cells (Figure 3). Deletion of type I collagen in myCAFs by using dual-recombinase pancreatic cancer mouse model (*Pdx1-Flp*; *frt-stop-frt-Kras^G12D/+^*; *Trp53^frt/frt^*; *Acta2-Cre*; *Col1a1^lox/lox^*) accelerates pancreatic cancer progression, decreases overall survival of mice, increases *Cxcl5* expression, and increases number of myeloid-derived suppressor cells (MDSCs) [21]. CXCR2 ligands are also produced by pancreatic tumor cells, recruiting MDSCs [21], but also activating CXCR2 signaling in CAFs. CAFs express CXCR2 ligands as well as CXCR2 [20]. CXCR2 signaling in CAFs causes activation of NF-κB signaling and secretes inflammatory cytokines and CXCR2 ligands [20]. Primary pancreatic cancer cells from *Ptf1a-Cre*; *lox-stop-lox-Kras^G12D/+^*; *Tgfbr2^lox/lox^* mice secrete CXCR2 ligands including CXCL1, CXCL2, and CXCL5 [22]. CXCR2 ligands induce *Ctgf* expression in CAFs via CXCR2 signaling [22]. Treatment with CXCR2 inhibitor Repertaxin or SB225002 inhibits *Ctgf* expression in CAFs (Figure 3) [22]. Blocking the paracrine activation of CXCR2 leads to attenuation of pancreatic tumor development, reduction of tumor angiogenesis, and prolongation of survival in *Ptf1a-Cre*; *lox-stop-lox-Kras^G12D/+^*; *Tgfbr2^lox/lox^* mice [22]. Another study shows that CAFs in metastasis (also called metastasis-associated fibroblasts) secrete CXCL8 and CCL2 promoting angiogenesis in metastasized pancreatic cancer [48]. On the contrary, it has been shown in another study that when pancreatic tumor cells derived from KC mice are orthotopically implanted in the pancreas, host global *Cxcr2* loss inhibits micro-vessel density in pancreatic tumors, but does not inhibit pancreatic cancer growth, and enhances liver metastasis [49]. Pancreas-specific deletion of *Cxcr2* in KC mice prevents oncogene-induced senescence, increases tumor proliferation, and decreases survival (*Pdx1-Cre*; *lox-stop-lox-Kras^G12D/+^*; *Cxcr2^lox/lox^*) [50]. The reason why the outcomes between the study with the CXCR2 inhibitor and with the global *Cxcr2* loss mouse model are different has not been fully answered.

Activated PSCs sequester CD8^+^ T cells to reduce their infiltration of the juxtatumoral compartment (less than 100 µm from the tumor) of pancreatic cancer [51]. Pancreatic cancer patients with high density of CD8^+^ T cells in the juxtatumoral compartment exhibit prolonged postsurgical survival [51]. Administration of all-trans retinoic acid (ATRA), which drives PSCs quiescent, increases numbers of CD8^+^ T cells in juxtatumoral compartments in KPC mice [51]. T cells are excluded where FAP-positive CAFs expressing CXCL12, also known as stromal cell-derived factor 1 (SDF1), are localized. CXCL12 plays a role in tumoral immunosuppression (Figure 3) [23]. Lack of NF-κB subunit *Nfkb1* in PSCs reduces CXC12 secretion, increases infiltration of CD8^+^ T cells, inhibits tumor growth, and improves host survival, evaluated by orthotopic co-injection experiments with primary tumor cells from KPC mice and PSCs [52]. CXCL12 is the ligand for the chemokine receptor CXCR4 (also known as Fusin or CD184) and CXCR7 (also known as atypical chemokine receptor 3, ACKR3) (Figure 3) [53]. Treatment with AMD3100 (plerixafor), a CXCR4 antagonist, attenuates tumor growth after co-injection of tumor cells from KPC mice and PSCs [52]. Another CXCR4 antagonist, BL-8040 (Motixafortide), in combination with programmed death 1 (PD-1, also known as CD279) antagonist pembrolizumab increases CD8^+^ T cell tumor infiltration and decreases tumor cell density (COMBAT trial, NCT02826486) [54]. It has been however shown that conditional knockout of *Cxcr4* in KPC mice (*Pdx1-Cre*; *lox-stop-lox-Kras^G12D/+^*; *Trp53^R172H/+^*; *Cxcr4^lox/lox^*) attenuates fibrogenesis and decreases α-SMA and PDGFR-α-positive cells but increases tumor size [55]. Further study is needed to answer why outcome between the study with CXCR4 inhibitor and with *Cxcr4* loss are inconsistent. CXCL12 binds CXCR7 with higher affinity than CXCR4 [53]. High CXCR7 expression is associated with shorter overall survival of pancreatic cancer patients. Patients with high expression of both CXCL12 and CXCR7 have even shorter overall survival than patients with high tumoral CXCL12 expression or CXCR7 expression alone [56]. In CXCL12-treated pancreatic cancer cells, CXCR4 antagonist AMD3100 does not inhibit migration and invasion, indicating CXCR7 promotes pancreatic cancer cell migration and invasion (Figure 3) [56]. CXCR7 promotes hepatic metastasis, but not orthotopic pancreatic cancer cell growth [56]. Whether *Cxcr4* deficiency can increase activity of CXCR7 signaling in pancreatic cancer has not been analyzed.

Depletion of focal-adhesion kinase (FAK) in the FSP1-positive cells in mice (*Fsp-Cre*; *Fak^lox/lox^*) increases breast and pancreatic cancer growth [24]. Low FAK expression in the stromal compartment is associated with shorter overall survival in human breast and pancreatic cancer patients [24]. *Fak*-depletion in CAFs promote secretion of several chemokines CCL6, CCL11, and CCL12. These chemokines activate chemokine receptors CCR1/CCR2 on cancer cells (Figure 3). The enhanced activation of protein kinase A (PKA) in tumor cells is required for reprogramming cellular metabolism. Exposure to *Fak*-depleted CAF cellular medium enhances glycolysis in tumor cells by upregulating glycolysis enzymes including pyruvate kinase, as well as glucose-6-phosphate dehydrogenase (G6PD) and 6-phosphogluconate dehydrogenase (6PGD) in the oxidative branch of pentose-phosphate pathway (PPP) [24]. Paracrine activation of CCR1 signaling has also been shown to be associated with regulatory T cells (Tregs). The CXCL12-CXCR4 signaling pathway can recruit Tregs [57]. Treg frequency correlates with metastasis, advanced tumor stage, high tumor grade, and shorter overall survival of pancreatic cancer patients [58]. To that end, depletion of Tregs may be a therapeutic strategy for pancreatic cancer. However, depletion of Tregs in KC mice (*Ptf1a-Cre*; *lox-stop-lox-Kras^G12D/+^*; *Foxp3^tm3(DTR/GFP)Ayr^*) induces reprogramming the fibroblast population, leading to a reduced number of α-SMA^+^ myCAFs; an increase in *Ccl3*, *Ccl6*, and *Ccl8* expression; increased myeloid cell infiltration; and accelerated pancreatic carcinogenesis [59]. CCR1 is the common chemokine receptor for the chemokines CCL3, CCL6, and CCL8, which are chemoattractants for myeloid cells [60]. CCR1 blockade with an inhibitor BX471 attenuates pancreatic carcinogenesis in *Ptf1a-Cre*; *lox-stop-lox-Kras^G12D/+^*; *Foxp3^tm3(DTR/GFP)Ayr^* mice [59].

## 6. Exosomes and Pro-Metastatic Niche in Pancreatic Cancer

Exosomes are a subset of cell-released, membranous-structured extracellular vesicles that also include microvesicles, microparticles, ectosomes, and apoptotic bodies [61]. Exosomes are of endosomal origin and secreted from various cell types including CAFs and cancer cells, and contribute to physiological processes, such as immune response and protein and RNA transport [62]. High *KRAS* mutation ratio (≥5%) in circulating exosomal DNA is associated with shorter progression-free survival and overall survival of pancreatic cancer patients [63]. Due to their endosomal origin, exosomes exhibit multiple proteins involved in the formation of multi-vesicular bodies (MVBs) such as annexins, Rab family GTPases, and endosomal sorting complexes required for transport (ESCRT) complex proteins [64]. Additional exosome protein markers include tetraspanins (CD9, CD63, CD81) and heat shock proteins (HSP60, HSP70, HSP90) [64]. A Dickkopf 1 (DKK1) receptor cytoskeleton-associated protein 4 (CKAP4) has been proposed as a biomarker, which is secreted with exsosomes from pancreatic cancer cells [65]. High expression of CKAP4 is detected in pancreatic cancer patient sera and in sera of pancreatic cancer cell-xenografted mice [65]. Blockade of the DKK1-CKAP4 binding by anti-CKAP4 monoclonal antibody inhibits xenograft tumor formation and metastasis of pancreatic cancer cells and extends survival of mice [65]. Glypican-1 (GPC1), a membrane-anchored protein that is overexpressed in pancreatic cancer, is present in cancer exosomes [66]. Pancreatic cancer patients exhibit higher GPC1^+^ circulating exosomes than in healthy donors, and elevated level of GPC1^+^ circulating exosomes is also observed in *Ptf1a-Cre*; *lox-stop-lox-Kras^G12D/+^*; *Tgfbr2^lox/lox^* mice [66]. GPC1 alone has an 82% sensitivity and 52% specificity for pancreatic cancer screening [67]. Multiparametric plasma extracellular vesicle profiling with five markers (named PDAC^EV^ signature) with EGFR, EPCAM, MUC1, GPC1, and WNT2 shows an 86% sensitivity and 81% specificity [67]. Another study suggests combined detection of exosomal GPC1, exosomal CD82, and serum CA19-9 for pancreatic cancer screening [68]. Treatment with GPC1 antibody conjugated with microtubule inhibitor monomethyl auristatin E inhibits pancreatic cancer in a pancreatic tumor xenograft mouse model [69]. GPC1 is also expressed on FAP-positive CAFs in pancreatic cancer [69]. GPC1 antibody conjugated with monomethyl auristatin E is delivered to the GPC1-expressing CAFs, leading to apoptosis of surrounding pancreatic cancer cells [69]. It has been shown that exosomes from CAFs increase pancreatic cancer cell survival and proliferation [70]. GW4869 is a neutral sphigomyelinase inhibitor, which blocks release of mature exosomes from MVBs [71]. Inhibition of exosome secretion from CAFs by GW4869 reduces pancreatic cancer cell survival [70].

Tumor-driven exosomes can prepare the pre-metastatic niche in distant organs. Integrin subtypes expressed on tumor-driven exosomes can predict the site of metastasis [72]. It has been demonstrated that macrophage migration inhibitory factor (MIF) is highly expressed in pancreatic cancer-derived exosomes. Knockdown of MIF in exosomes prevents pre-metastatic niche formation in the liver and metastasis [73]. Integrin α6β4 and α6β1 associated with lung metastasis and integrin αvβ5 correlates with liver metastasis [72]. Protein kinase D1 (PRKD1) reprograms pancreatic acinar cells to a ductal phenotype and drives progression to intraepithelial neoplasia (PanIN) [74]. The expression of PRKD1 is, however, downregulated in human pancreatic cancer [75]. Conditional deletion of *Prkd1* accelerates pancreatic tumorigenesis, drives lung metastasis, and enhances secretion of extracellular vesicles (*Ptf1a-Cre*; *lox-stop-lox-Kras^G12D/+^*; *Prkd1^lox/lox^*) [75]. *Prkd1*-deficiency in pancreatic cancer cells increases α6β4 loading into extracellular vesicles that requires CD82 [75]. Exosomes from mutant p53 (R270H or R175H)-expressing cells increase diacylglycerol kinase α (DGKα)-dependent trafficking of integrin and cell migration [76]. Exosomes from mutant p53-expressing cells act on CAFs, leading to remodeling of ECM to support tumor cell migration and invasion [76]. Exosomes can be also considered as a carrier of therapeutic reagents. Treatment with exosomes carrying short interfering RNA or short hairpin RNA specific to Kras^G12D^ suppresses pancreatic cancer progression and improves survival of KPC and *Ptf1a-Cre*; *lox-stop-lox-Kras^G12D/+^*; *Tgfbr2^lox/lox^* mice [77].

## 7. Conclusions

Recent studies have demonstrated that a large number of factors, including growth factors, hedgehog molecules, inflammatory cytokines, and chemokines derived from both cancer-associated fibroblasts and tumor cells, act as key autocrine, paracrine, and reciprocal mediators in signal transduction networks. Cancer-associated fibroblasts play an important role, not only in the tumor microenvironment but also in secretion, regulation of exosomes, and in the preparation of the pre-metastatic niche in distant organs. Exosomes can be used as a carrier of therapeutic reagents for pancreatic cancer. A broad and better understanding of the interactions between cancer-associated fibroblasts and tumor cells is important for developing novel therapeutic strategies that improve the outcome of pancreatic cancer patients.

## Figures and Tables

**Table 1:** Overview of autocrine, paracrine, and paracrine-reciprocal factors secreted from cancer-associated fibroblasts and tumor cells.

Factor	Source	Mode of Action	Functional Relevance	Reference
CTGF	CAFs, tumor cells from KPC mice	Paracrine	Act on CAFs and tumor cells	[11]
	Pancreatic cancer cells	Paracrine	Promotes proliferation, adhesion, migration of PSCs	[17]
HGF, IL-6	CAFs, tumor cells	Paracrine	Act on CAFs and tumor cells	[13]
SHH	Pancreatic tumor cells	Paracrine	Induces expression of IGF1 and GAS6 in PSCs	[18]
IGF1	PSCs	Paracrine- reciprocal	Activates IGF1R signaling	[18]
GAS6	PSCs	Paracrine- reciprocal	Activates AXL signaling	[18]
IL-1α	Pancreatic tumor cells	Paracrine	Activates NF-κB signaling and expression of LIF in iCAFs	[19]
LIF	iCAFs	Autocrine	Activates JAK/STAT signaling and establishes a positive feedback loop by upregulating IL-1R1	[19]
LIF	iCAFs	Paracrine (-reciprocal)	Activate STAT3 signaling in cancer cells	[19]
KRAS-driven factors (unknown)	Pancreatic cells	Paracrine	Act on CAFs inducing secretion of CXCR2 ligands and CXCR2 expression	[20]
CXCR2 ligands	CAFs	Autocrine	CXCR2 signaling in CAFs	[20]
CXCR2 ligands	Pancreatic tumor cells	Paracrine	Recruit MDSCs	[21]
CXCR2 ligands	Pancreatic cancer cells from *Ptf1a-Cre; lox-stop-lox-Kras^G12D/+^; Tgfbr2^lox/lox^* mice	Paracrine	Induce *Ctgf* expression in CAFs	[22]
CXCL12	CAFs	Paracrine	Immunosuppression	[23]
CCL6, CCL11, CCL12	*Fak*-depleted CAFs	Paracrine	Activate CCR1/CCR2 on cancer cells	[24]

AXL: anexelenko, CAF: cancer-associated fibroblast, CTGF: connective tissue growth factor, CXCR: C-X-C motif chemokine receptor, GAS: growth arrest-specific, HGF: hepatocyte growth factor, iCAF: inflammatory CAF, IGF: insulin growth factor, IL: interleukin, IL-1R1: IL-1 receptor type 1, KPC: *Pdx1-Cre*; *lox-stop-lox-Kras^G12D/+^*; *Trp53^R172H/+^*, LIF: leukemia inhibitory factor, MDSC: myeloid-derived suppressor cell, NF-κB: nuclear factor kappa-light-chain-enhancer of activated B cells, PSC: pancreatic stellate cell, SHH: Sonic hedgehog.
